# Supplementary material for: Magnetism and exchange interaction of small rare-earth clusters; Tb as a representative
Source: Sci Rep. 2016 Jan 22;6:19676. doi: 10.1038/srep19676 (PMC4726341; doi:10.1038/srep19676)
Supplement: Supplementary Information [file srep19676-s1.doc]

Magnetism and exchange interaction of small rare-earth clusters; Tb as a representative

Lars Peters, Saurabh Ghosh, Biplab Sanyal, Chris van Dijk, John Bowlan, Walt de Heer,
Anna Delin, Igor Di Marco, Olle Eriksson, Mikhail I. Katsnelson, Börje Johansson
and Andrei Kirilyuk

**Supplementary information**

***Magnetic anisotropy***

In the magnetic deflection experiments, apart from the average deflection amplitude proportional to the magnetic moments, some of the cluster beam profiles show considerable broadening. Moreover, in these cases, some of the clusters deflect in the direction opposite to the magnetic field, as the inset in Fig. S1 shows. Even though qualitatively similar to the rigid-rotor model [S1], the broadening can also be explained by the internal spin-lattice relaxation mechanism [S2]. In contrast, “negative” deflection can only happen if the magnetic moments are “locked” with respect to the lattice. For this to happen the anisotropy energy should be sufficiently large to overcome the thermal fluctuations of the order of k_B_T. A simple estimate for the experimental temperature of 77 K and fly-time of about 1 ms gives a lower limit for the anisotropy energy of at least 10 meV/atom. To give a perspective, the famous FePt L1_0_ alloy possesses magnetic anisotropy of 2.8 meV/atom [S3].

FIG. S1. Magnetic anisotropy energy of Tb clusters, showing values in agreement with the experiments. The anisotropy energy surfaces are also shown for three cluster sizes.

Bulk Tb is known for its very large uniaxial anisotropy along the hexagonal axis, with an anisotropy energy at low temperatures of about 1 meV per atom. How this anisotropy is modified for the clusters can be addressed by theory.

Magnetic anisotropy energies (magnetocrystalline anisotropy) and orbital moments were calculated by including spin-orbit coupling in the Kohn-Sham Hamiltonian. The details are described in Ref. S4. These properties were calculated with the optimized geometries including spin-orbit interaction. The fully self-consistent calculations resulted in slight canting of moments as shown in Fig. 2. For each cluster, magnetic anisotropy energy was calculated as the difference between fully self-consistently calculated total energies along the easy and hard axes of magnetization.

The calculated changes in energy range from 5 to 12 meV/atom in clusters from n=4 to 12, see Fig. S1, which is qualitatively in agreement with the experimental results. One should however note that the single-particle description used in the LDA+U approach applied in this part of our study may not be optimal for describing the spin-orbit induced effects for *f* shells. First, a non-integer 4*f* occupation is observed and second the true many-electron picture for atomic multiplets is missing. However, the general trend of an enhanced magnetic anisotropy of the clusters is captured by this calculation. For all these calculations the program VASP was used, see ‘Methods: Theoretical methods and computational details II’ for more technical details.

**References**

[S1] Bertsch, G. F. and Yabana, K. Cold cluster ferromagnetism. *Phys. Rev. A* **49**, 1930-1932 (1994)

[S2] Xu, X., Yin, S., Moro, R. and de Heer, W. A. Magnetic Moments and Adiabatic Magnetization of Free Cobalt Clusters. *Phys. Rev. Lett.* **95**, 237209 (2005)

[S3] Weller, D. et al. High K_u_ materials approach to 100 Gbits/in^2^. *IEEE Trans. Magn.* **36**, 10-15 (2000)

[S4] Blonski, P., Dennler, S. and Hafner, J. Strong spin–orbit effects in small Pt clusters: Geometric structure, magnetic isomers and anisotropy. *J. Chem. Phys*. **134**, 034107 (2011)
